# Are Farm-Reared Quails for Game Restocking Really Common Quails (*Coturnix coturnix*)?: A Genetic Approach

**DOI:** 10.1371/journal.pone.0039031

**Published:** 2012-06-12

**Authors:** Ines Sanchez-Donoso, Carles Vilà, Manel Puigcerver, Dalius Butkauskas, José Ramón Caballero de la Calle, Pablo Antonio Morales-Rodríguez, José Domingo Rodríguez-Teijeiro

**Affiliations:** 1 Conservation and Evolutionary Genetics Group, Doñana Biological Station, EBD-CSIC, Seville, Spain; 2 Animal Biology Department, University of Barcelona, Barcelona, Spain; 3 Experimental Sciences and Mathematics Didactics Department, University of Barcelona, Barcelona, Spain; 4 Laboratory of Molecular Ecology, Nature Research Centre, Vilnius, Lithuania; 5 Sciences, Agroforestry Technology and Genetics Department, University of Castilla-La Mancha, Ciudad Real, Spain; North Carolina State University, United States of America

## Abstract

The common quail (*Coturnix coturnix*) is a popular game species for which restocking with farm-reared individuals is a common practice. In some areas, the number of released quails greatly surpasses the number of wild breeding common quail. However, common quail are difficult to raise in captivity and this casts suspicion about a possible hybrid origin of the farmed individuals from crosses with domestic Japanese quail (*C. japonica*). In this study we used a panel of autosomal microsatellite markers to characterize the genetic origin of quails reared for hunting purposes in game farms in Spain and of quails from an experimental game farm which was founded with hybrids that have been systematically backcrossed with wild common quails. The genotypes of these quail were compared to those of wild common quail and domestic strains of Japanese quail. Our results show that more than 85% of the game farm birds were not common quail but had domestic Japanese quail ancestry. In the experimental farm a larger proportion of individuals could not be clearly separated from pure common quails. We conclude that the majority of quail sold for restocking purposes were not common quail. Genetic monitoring of individuals raised for restocking is indispensable as the massive release of farm-reared hybrids could represent a severe threat for the long term survival of the native species.

## Introduction

Restocking has become one of the most commonly used practices for the conservation and management of endangered and game species. One important reason for restocking is to supplement fisheries and game bags in order to increase productivity or maintain recreational activities [1]. In some cases this may threaten local gene pools [2]. Restocking for hunting purposes has been carried out with diverse species such as deer [3], marmot [4], wild boar [5], waterfowl [6] and different Galliform species [7], [8], including partridges [9]–[11], pheasants [12] and quails [13]–[15].

The common quail (*Coturnix coturnix*) is a migratory Galliformes species distributed across Eurasia during its breeding period [16] and currently has an unfavourable conservation status in Europe due to a large historical decline [17]. Even so, the common quail is a popular game species with an important socio-economic impact. Only in Spain, more than 1,300,000 quails have been hunted annually during the period 1973–2010 (*Yearbook of Agro-alimentary Statistics* of the Spanish Ministry of Agriculture, Fishing and Food). Restocking with farm-reared quails to increase bag numbers is a very common practice in several European countries, such as Italy [18], Greece [14], [19], The Republic of Serbia, Montenegro, Romania and Spain [13], [15]. According to Guyomarc'h [16], 430,000–480,000 released quails were shot in France during the 1983–1984 hunting season, corresponding to 67%–75% of the 640,000 total individuals hunted. In Catalonia (Northeast Spain), an average of more than 68,000 farm-reared quails have been released annually during the period of 1990–2006 (Hunting order plans of the Regional Government of Catalonia). Taking into account that the estimated wild male quail population in Catalonia ranges between 5,347 and 20,847 individuals [20], the number of quails restocked greatly exceeds the breeding population.

However, rearing common quails in captivity for restocking is difficult because of their restlessness [21]. This has led to some concern about the genetic origin of farm-reared quails. A possible explanation for the great reproductive success in farms is that those individuals could be of hybrid origin, resulting from crosses between wild common quails and domestic Japanese quails (*Coturnix japonica*). The Japanese quail is sister species to the common quail and is distributed across eastern Asia [22]. For several centuries Japanese quails have been bred in captivity and selected for meat and egg productivity [23]. These domestic Japanese quail first entered Europe (France and Italy) in the 1950 s [24], [25]. As a result of the selection for life in captivity, these birds have lost their migratory restlessness [26], show some reluctance to move and fly, and have lower anti-predatory instinct.

These differences in behaviour suggest that restocking with farm-reared domestic Japanese quails or hybrids between the two species (using this term to refer to all admixed individuals and not just first generation hybrids) would result in a conservation problem. In fact, several authors have warned about the risk that restocking can represent for common quails when carried out with domestic Japanese quails and hybrids [14]–[16], [27], [28]. It entails a potential threat to the native common quail because it could lead to the introgression of domestic Japanese quail alleles and a subsequent loss of migratory behaviour and decline in fitness in the native common quail population [16], [29]. Individuals that do not migrate or that show low mobility could be more exposed to adverse climatic conditions, loss of habitat after the harvesting of cereal crops (the usual habitat of the species), predation, or a lack of food resources [16]. Thus, the arrival of maladaptive genes from domestic Japanese quail could reduce the survival and adaptive potential of wild common quail.

This conservation concern seems to be a real one: hybrid individuals have already been detected in common quail breeding areas in different European countries such as Portugal, France, Italy and Spain [13]–[16]. Moreover, recent field experiments with released radio-tagged farm-reared individuals of hybrid origin have shown that they can mate with wild common quails and produce fertile offspring (unpub. data) as happens in captivity [30]. Other closely related native Galliformes, the red-legged partridge (*Alectoris rufa*) [31], [32] and the rock partridge (*A. graeca*) [33], are threatened by hybridization resulting from game restocking with sister species or hybrids. As a result of the concern about hybridization, the release of Japanese quails and hybrids is illegal in Spain, Portugal, France and Greece. However, the diagnosis of the specific origin of farmed quails used for restocking is usually based on their morphology despite the difficulty in unequivocally differentiating between pure common quails and admixed individuals on the basis of their phenotype ([34] in [14]). Thus, genetic analyses are needed to assess species identity of restocked farm-reared individuals.

The aim of this study was to identify the genetic origin of quails reared for restocking and hunting purposes in five different Spanish game farms by using a panel of nuclear microsatellite markers. We also analysed quails from an experimental farm that managed to reduce the genetic contribution of the founders, which had domestic Japanese quail ancestry, by crossing farm-born individuals with wild common quails. We hypothesize that a large proportion of the released quails are of hybrid ancestry due the easy breeding of domestic Japanese quails in captivity. If this is the case, the release of these birds could negatively affect the long term survival of natural common quail populations.

## Materials and Methods

### Ethics Statement

All work related with animals in this study has been conducted according to the guidelines of the Federation of European Laboratory Animal Science Associations (FELASA). It fulfills the ethic recommendations of the European Union and the Spanish legislation and has been approved by the Ethics Committee on Animal Experimentation from the University of Barcelona and from the University of Castilla-La Mancha.

### Samples origin and collection

One hundred and fifty-two quails were sampled for this study. They were collected from four different origins (Table 1). They consist of: 1) Males and females randomly sampled from five different Spanish game farms in 2009 and 2010, purchased for restocking and hunting purposes. 2) Quails from an experimental farm from the University of Castilla-La Mancha (Spain) managed for about 20 years to reduce the genetic contribution of founders. These individuals are descendants of crosses between admixed females with wild common quail males whose offspring has been backcrossed with wild common quail males in successive generations. 3) Wild quail males captured (see [15]) during 1996–2009 around Seville (South Spain), Barcelona (Northeast Spain) and Drenthe (The Netherlands) which were identified as common quails on the basis of their song, phenotype and preliminary genetic analyses. 4) Domestic Japanese quails from two laboratory lines from the Laboratory of Molecular Ecology of the Institute of Ecology of the Nature Research Centre, Vilnius University (Lithuania) [35], and from four different Spanish meat farms.

**Table 1:** Quail samples studied.

Group	N_G_	Sampling origin	N
Game farm quails	52	Game farm 1	13
		Game farm 2	7
		Game farm 3	20
		Game farm 4	6
		Game farm 5	6
Experimental quails	19	Experimental farm	19
Wild common quails	42	Seville (S Spain)	5
		Barcelona (NE Spain)	25
		Drenthe (The Netherlands)	12
Domestic Japanese quails	39	Meat farm (4 farms, 4 samples from each)	16
		Laboratory lines (2 lines with 9 and 14 samples)	23
**Total**			152

N_G_: number of individuals per group. N: number of individuals per sampling origin.

Blood (100 µl) or muscle samples were individually stored at −20°C in 95% ethanol until DNA was extracted using DNeasy Blood & Tissue Kit (Qiagen) following manufacturer's protocols.

### Typing of microsatellite loci

Individuals were genotyped for 11 unlinked autosomal microsatellite loci originally developed for Japanese quail [36], [37]: GUJ0001, GUJ0017, GUJ0028, GUJ0039, GUJ0044, GUJ0057, GUJ0065, GUJ0074, GUJ0085, GUJ0093 and GUJ0097. Loci were amplified by polymerase chain reaction (PCR). While some markers were PCR-amplified in a multiplex, others were amplified separately and subsequently pooled before electrophoresis. Detailed protocols are available upon request. All PCR products were electrophoresed on an ABI 3730 sequencer (Applied Biosystems) following manufacturer's protocols. Alleles were sized and scored using the software GeneMapper v3.5 (Applied Biosystems).

### Analysis of microsatellite data

To measure genetic variation, average number of alleles per locus and allelic richness (mean number of alleles per locus corrected for minimum sample size, in this case nine successfully genotyped individuals per population and locus) [38] were calculated using FSTAT version 2.9.3.2 [39]. In order to measure the marker informativeness we calculated the Polymorphic Information Content (PIC) [40], which takes into account the number of alleles per locus and the frequency of these alleles, using EXCEL MICROSATELLITE TOOLKIT 3.1.1 [41]. We used the same software to calculate observed (H_O_) and expected (gene diversity, H_E_) heterozygosities [42].

Patterns of genetic differentiation were visualized by plotting the individuals in a two-dimensional space according to their microsatellite composition, independently of any *a priori* classification, using a factorial correspondence analysis (FCA) in GENETIX [43].

To identify genetically distinct clusters present in the data we used a Bayesian clustering procedure implemented in STRUCTURE 2.3.2 [44]. STRUCTURE identifies the number (*K*) of genetically distinct clusters that maximizes the likelihood of the data and estimates, for each individual, the fraction of the genome (q) that belongs to each one of the clusters. Analyses were performed using all individuals under the “admixture model” (as each individual may have ancestry in more than one parental population), with correlated allele frequencies and without population or sampling location information (USEPOPINFO and LOCPRIOR inactives). Simulations were run for 100,000 steps following a burn-in period of 30,000 steps, considering values of *K* ranging between two and 10, and were replicated five times [45] after verifying that results did not vary significantly with longer runs of iterations. Likelihood values were observed to converge during the runs. For *K* = 2, for each individual we estimated the 90% probability interval for the proportion of membership to each cluster (q). The best value of *K* was chosen following the method proposed by Evanno et al. (2005) [46], with STRUCTURE HARVESTER [47], which takes into account the rate of change in the log likelihood between successive *K* values. Each individual was assigned exclusively to one of the inferred clusters when its q to that cluster was equal or larger than a threshold corresponding to the minimum value observed among the non-admixed individuals used as reference (see below). Alternatively, individuals that showed lower q values for all clusters could not be assigned exclusively to one of them and were considered putatively admixed.

After confirming with STRUCTURE that none of the wild common quails and domestic Japanese quails had a genome that seemed admixed, we considered them purebred and used them as reference in analysis with NEWHYBRIDS 1.1 [48]. With this software, we computed the posterior probability (P) for each individual to belong to each of the following genotypic classes: parental purebred 1 (P_1_), parental purebred 2 (P_2_), first generation hybrid (F_1_), second generation hybrid (F_2_, offspring of crosses between F_1_ hybrids), backcross of F_1_ with P_1_ (Bx_1_) and backcross of F_1_ with P_2_ (Bx_2_). Posterior distributions were evaluated after running five independent analyses to confirm convergence, starting with different random seeds, for 10^5^ Monte Carlo Markov Chain iterations after 10^4^ burn-in steps, without using prior allele frequency information. Analyses were run for four combinations of prior distributions (JEFFREYS or UNIFORM for Theta and Pi) to explore the robustness of the results, as recommended by the software authors [48]. The affinity of an individual to the genotype classes was assessed by its posterior probability values (P): those that showed P≥0.85 to one genotype class were assigned to that class; if no value reached 0.85, but the sum of all hybrid classes was above this threshold, individuals were identified as hybrids of unknown generation [45]; individuals whose origin could not be identified under these criteria were left unclassified.

## Results

### Loci and population genetic characteristics

All 152 individuals studied were successfully genotyped at seven or more of the 11 markers, and more than 95% of them were typed for eight or more loci. All loci were polymorphic in the four groups of quails studied. A total of 224 alleles were found, 145 of which were exclusive to the reference wild common quails and eight to the reference domestic Japanese quails (excluding individuals from game and experimental farms), implying great power for hybrid identification.

Domestic Japanese quails showed the lowest average number of alleles per locus, allelic richness and PIC while the highest values were found in wild common quails (Table 2). Values for game and experimental farm quails were intermediate. Observed heterozygosity was higher than expected in experimental farm quails (p = 0.0003). On the other hand, observed heterozygosity was lower than expected in domestic Japanese quails (p = 0.0012) as could be expected considering that the samples originated from separate breeding lines.

**Table 2:** Genetic diversity for each group of samples.

Group	N	Average number of alleles	Allelic richness	PIC	H_E_	H_O_
Game farm quails	52	14.00	7.65	0.80	0.83	0.80
Experimental farm quails	19	8.64	6.93	0.77	0.82	0.84**
Wild common quails	42	17.73	9.82	0.87	0.90	0.90
Domestic Japanese quails	39	5.27	4.11	0.59	0.66	0.60*

N: number of individuals. PIC: Polymorphic Information Content [40]. H_E_: expected heterozygosity [42]; H_O_: observed heterozygosity [42]. Significant differences between H_E_ and H_O_ are indicated by * (p≤0.05) and ** (p≤0.001).

### Population differentiation

**Figure 1:**
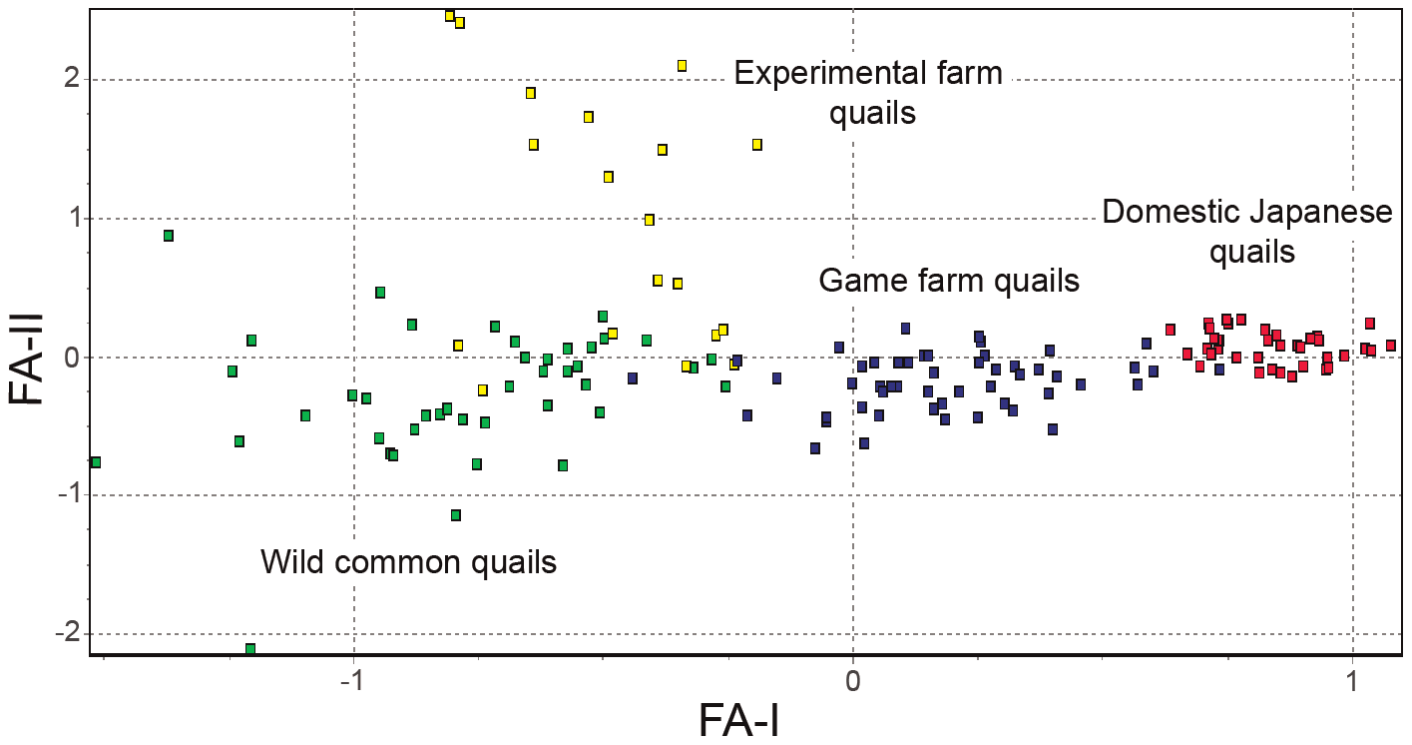
Factorial correspondence analysis. Green: wild common quails; red: domestic Japanese quails; blue: game farm quails; yellow: experimental farm quails.

Wild common quails appeared completely separate from domestic Japanese quails along the first factorial component (FA-I) of the FCA (Figure 1). Game farm quails occupied an intermediate position between common quails and domestic Japanese quails along the same axis showing almost no overlap with either of them. Individuals from the experimental farm occupied the same range of values compared to wild common quails along the first axis, but showed a clear separation along the second axis (FA-II), with some individual values overlapping the range observed for wild common quails.

**Figure 2:**
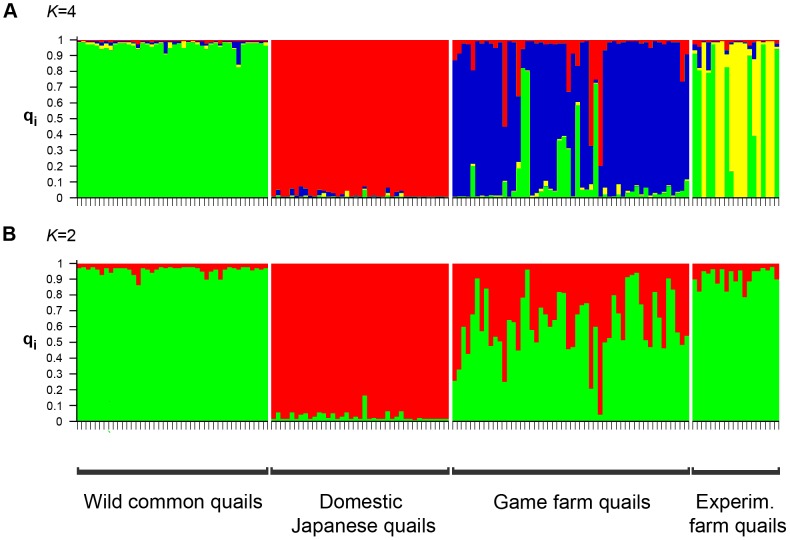
Clustering of individual genotypes into *K* = 4 (A) or *K* = 2 (B) clusters according to STRUCTURE. Each vertical bar represents one individual and clusters are represented by colours. The extent of the colours within each column represents the individual proportion of membership (q) to each one of the clusters.

According to the approach of Evanno et al. (2005) [46], the total sample could be subdivided in four clusters (Figure 2). Likelihood values converged during the runs and results did not change across replicates. Common quails and domestic Japanese quails were completely separated in two different clusters. All wild common quails (N = 42) were unequivocally assigned to cluster 1 (C_1_), with q_1_≥0.84, while all domestic Japanese quails (N = 39) were assigned to cluster 2 (C_2_), with q_2_≥0.92. Considering these results, we established that q values under a threshold value of 0.84 could be suggesting admixed ancestry. Of the game farm quails (N = 52), 73% grouped in a separate cluster (C_3_) while 27% appeared as admixed. Experimental farm quails (N = 19) split between their own cluster (C_4_) (48%), and C_1_ (26%) (with common quails), while 26% of them were identified as admixed. These results show that none of the game farm quails were assigned to the same cluster as common quails, and this was also the case for 74% of the experimental farm quails.

### Hybrid identification

The partition of the sample in four groups was the result of the relative isolation between the different groups. However, since the sample included two evolutionarily distinct lineages (common and Japanese quails) and perhaps admixed individuals, we also investigated the partition of the sample into two groups (*K* = 2). The likelihood values converged during the runs and results did not change between replicates. Common quails and domestic Japanese quails appeared completely differentiated in separate clusters, as in the previous analysis (Figure 2). All wild common quails (N = 42) were unequivocally assigned to cluster 1 (C1), with q_1_≥0.87, while all domestic Japanese quails (N = 39) were assigned to cluster 2 (C_2_) with q_2_≥0.83 (Figure 2). In this case and considering these results, we established the value of 0.83 as the threshold below which individuals could be classified as admixed. Forty-three of 52 (83%) game farm quails had admixed genotypes, eight (15%) clustered in C_1_ with common quails and one (2%) with domestic Japanese quails in C_2_. Sixteen (84%) of the experimental farm quails clustered in C_1_, while the remaining three individuals (16%) had admixed genotypes (Figure 2).

**Figure 3:**
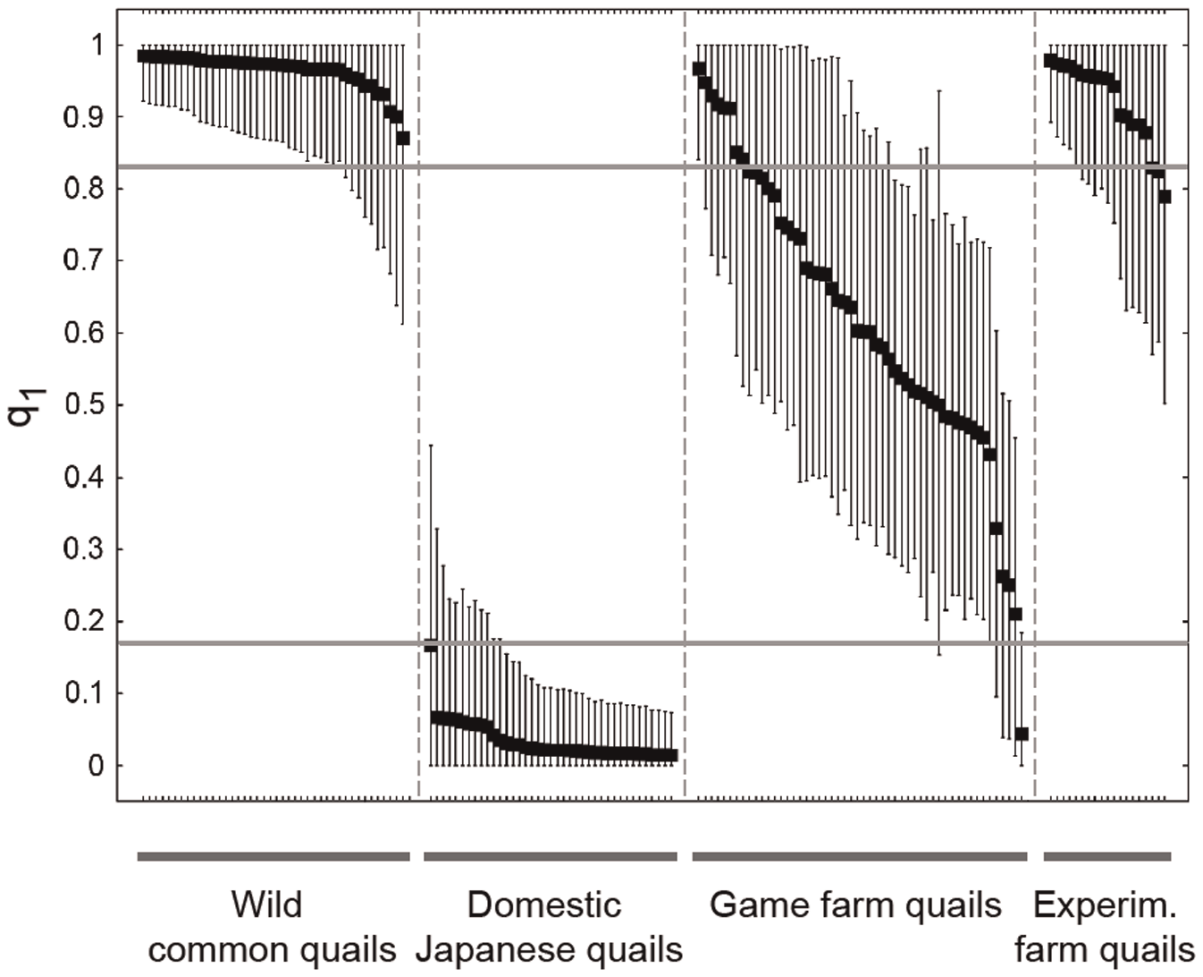
Individual proportion of membership to cluster 1 (q_1_) and 90% individual probability intervals according to STRUCTURE for *K* = 2. The value of q_1_ indicates membership to the same cluster as wild common quails. Individuals are sorted by group and by q_1_ value. Probability intervals excluding 0 and 1 are indicative of admixed ancestry. Horizontal lines indicate threshold values for q used for a first separation of pure and admixed individuals (see text).

We also evaluated the width of the 90% probability interval for each q for *K* = 2. While none of the wild common quails or domestic Japanese quails had probability intervals that excluded the possibility of being pure (q_1_ = 1 or 0, respectively; Figure 3), purebred ancestry could be excluded in 71% of the game farm quails. On the contrary, probability intervals did not exclude the possibility of being a pure common quail for any of the individuals from the experimental farm (Figure 3).

**Figure 4:**
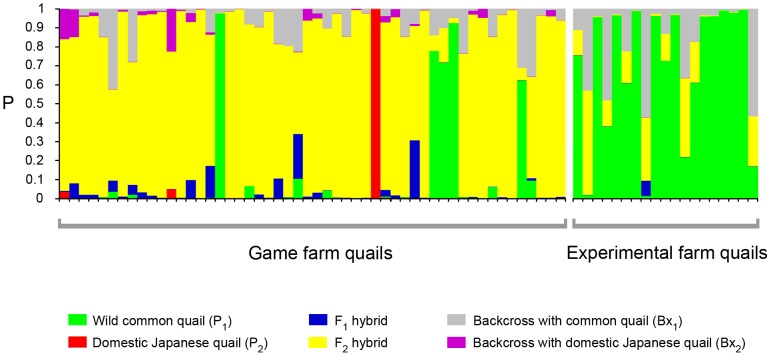
Individual genotype classification according to NEWHYBRIDS. Each vertical bar represents one individual. Each colour represents the posterior probability (P) of each individual to belong to the six different genotypic classes. Game farm and experimental farm individuals are sorted as in Figure 2.

After defining wild common quails and domestic Japanese quails as reference purebred groups, we used NEWHYBRIDS to identify the origin of all farm quails. More than 95% of wild common quails and domestic Japanese quails showed P≥0.85 to their genotypic class (P_1_ and P_2_, respectively), independently of the prior combinations used. This confirmed that these samples could be used as reference groups. Game farm individuals that were classified as F_2_ hybrids using a Jeffreys distributed prior for Theta were classified as domestic Japanese quails using a uniform prior. This last classification was considered unlikely due to the previously known information regarding the management of game farms (see discussion) and due to the results obtained with STRUCTURE when *K* = 2. For this reason, we only took into account results obtained using Jeffreys prior for Theta (individual classification did not show significant differences depending on the prior used for Pi). The analyses indicated that 46 out of 52 (88%) game farm quails were admixed (Figure 4). Thirty of these 46 could be identified as F_2_ hybrids, while 16 of them were hybrids of unknown generation (probably indicating older admixture). Three of the game farm quails (6%) could not be classified into a unique class, two were assigned as common quails and one as domestic Japanese quail. On the other hand, 10 out of 19 of the experimental farm quails (53%) were identified as common quails, seven (37%) could not be classified and two (10%) were hybrids of an unknown generation.

Comparing the results regarding game farm and experimental farm quails obtained with STRUCTURE for *K* = 2 and with NEWHYBRIDS, we observed assignment inconsistencies in only three individuals. For the three cases (three game farm quails) individuals were classified as common quails by STRUCTURE but as hybrids of unknown generation by NEWHYBRIDS. This is likely a result from ancient admixture with multiple generations of backcrossing with pure common quails. While all individuals were classified as either pure or admixed by the criteria that we defined in STRUCTURE, the classification with NEWHYBRIDS was more conservative and some of the individuals were left unclassified.

## Discussion

Our results showed that each one of the groups of quails studied had a unique identity that allowed its separation in genetic analyses. Thus, wild common quails, domestic Japanese quails, game farm quails and experimental farm quails formed four well separated groups, as suggested by STRUCTURE (Figure 2). Among these, wild common quails had the largest genetic diversity, as should be expected since the other groups represent smaller captive lineages that were more or less reproductively isolated from each other. Probably, the arrival of domestic Japanese quails from Asia into Europe did not involve many different lines [24], [25]. This bottleneck could have produced the low genetic diversity present in the domestic Japanese quail lines analysed. Similarly, the number of breeders in game farms is expected to be relatively small. On the other hand, domestic Japanese quails do not constitute a uniform population but are fragmented (with separate meat and laboratory lines, for example), as shown by the difference between observed and expected heterozygosity resulting from a Wahlund effect [49]. The opposite pattern is observed in the experimental farm, where outbred matings are regularly imposed.

The differentiation was largest between wild common quails and domestic Japanese quails since both groups appeared in the two extremes of the first axis of the FCA without any overlap (Figure 1). All STRUCTURE analyses clustered them in separate groups, including those with *K* = 2, which allowed separating the two evolutionary lineages. Also, a large number of alleles were not shared between the two groups, although this can be greatly affected by the sample size and by the fact that domestic Japanese quails were represented by inbred lines. This clear separation for the studied markers facilitates the identification of admixed individuals, overcoming the difficulties derived from their similar phenotypes.

Our analyses suggest that, contrary to the claims commonly expressed by farm managers, at least 85 to 90% (depending on the approach used) of the analysed game farm quails purchased for restocking purposes were not pure common quails and showed obvious signs of admixture with domestic Japanese quails. This estimate is based on the assumption that the reference individuals indeed represent purebred common and domestic Japanese quails. However, it is possible that some of them could have slightly admixed ancestry. Nevertheless, if this was the case, our numbers would be an underestimate of the proportion of admixed individuals in game farms, and the real proportion would be even larger.

This large proportion of admixed individuals does not seem exclusive to Spanish game farms. Previous genetic studies included a few game farm-reared quails and suggested hybrid origin in Portugal [50], France, Italy and Spain [14]. Most of the time, farm managers avoid revealing their procedures but indirect reports have suggested that they may often interbreed individuals of hybrid origin for many generations. We investigated the probability of farm individuals being pure or offspring of two generations of intercrossing or less (F_1_, F_2_ or a backcross of F_1_ to one of the two parental classes). It is likely that many of the individuals studied are the result of a larger number of generations of intercrossing, but in order to estimate the ancestry of each individual with higher precision we would require a larger number of markers with high PIC, which would allow a better separation between hybrid classes [45]. Nevertheless, our results clearly show the admixed ancestry of the studied game farm birds.

The analyses with NEWHYBRIDS indicated that, depending on the priors used, most game farm individuals were classified as domestic Japanese or F_2_ hybrids. We assumed that identification as F_2_ was more likely because of the results obtained with STRUCTURE with *K* = 2 and because of the mating strategies that may have been taking place in game farms to obtain individuals for hunting purposes [16]. Domestic Japanese quails and common quails [26], [51] could have been crossed, most likely domestic Japanese females with wild common quail males trapped in the field [50], and the offspring could have been subsequently intercrossed. With this procedure, fertile hybrids [30] may easily be obtained showing a “wilder” instinct, flying better and being smaller than the domestic Japanese quails [16], and thus being more attractive for sportive hunting. However, these hybrids inherit the reduced restlessness of domestic Japanese quails ([34] in [14]). Among the game farm quails analysed, between 4% and 15% were identified as common quails, depending on the approach used. This could reflect the occasional introduction of wild quails into the captive populations to act as breeders in order to decrease inbreeding and genetic load.

In the case of the experimental farm, common quail males have been regularly introduced to the farm breeding population during the last 20 years. In the last 10 years (about 10 generations) only females that are descendants of a common quail father have been used as breeders. Consequently, we expect a high frequency of common quail alleles in the genomes of quails from the experimental farm: 53 to 84% (depending on the methodological approach used) of the birds were classified as common quail. This implies that, after 20 generations of experimental crossing, between 10% and 16% of them are still identified as hybrids and 37% cannot be classified into any of the genotypic classes. In addition, we expect all individuals from this farm to carry Japanese quail mitochondrial DNA (see also [14]). The management strategy implemented in the experimental farm, trying to obtain individuals genetically more similar to the common quail than individuals from ordinary game farms, achieves its goal. However, we do not have any information about the possible cytonuclear conflicts deriving from the different origin of their mitochondrial and nuclear genomes, or about the relative fitness, behaviour and survival of these individuals in the wild compared to pure common quails. We do not know to which extent this management could minimise the impact of released admixed individuals on the native populations.

Due to the concern about the risk of restocking hybrid farm-reared quails, several European countries and regions have banned or regulated restocking practices (Portugal and France since 2002, Greece since 2007). In Spain, although different regions have different policies, the national law allows restocking with common quail, but not with Japanese quail or hybrids (Spanish Law 42/2007, Natural heritage and Biodiversity). Even so, many farm-reared individuals are still being released assuming that, in fact, they are pure common quails. Authorities allow quail releases trusting on the diagnosis of veterinarians that identify them as common quails on the only basis of their phenotype despite the fact that this identification method is usually ambiguous ([34] in [14]). Genetic analyses should be required to certify the origin of individuals used for restocking. Similarly, there is a need for an extensive survey of the diversity in wild quails to assess the impact that these releases may have had across its distribution range.
